# Origin of Fresnel problem of two dimensional materials

**DOI:** 10.1038/s41598-019-54338-0

**Published:** 2019-11-28

**Authors:** Xiaodong Wang, Bo Chen

**Affiliations:** State Key Laboratory of Applied Optics, Changchun Institute of Optics, Fine Mechanics and Physics, Chinese Academy of Sciences, Changchun, 130033 China

**Keywords:** Surfaces, interfaces and thin films, Optical properties and devices, Two-dimensional materials

## Abstract

Reflectance, transmittance, and absorption of materials are also known as materials’ Fresnel problem. It is widely accepted that Interface model can be utilized to solve Fresnel problem of two dimensional materials. Here, we question the validity of Interface model. Theoretical and experimental results of two dimensional materials are analyzed, and theoretical optical response of two dimensional materials is derived based on thin film model. A new simple, approximate formula of 4*πnkd/λ* is proposed for calculation of absorption of two dimensional materials. It is found that, in essence, Interface model is a kind of approximate style of thin film model, the main difference between two models is term of (*n*^2^ − *k*^2^) at normal incidence. A significant error is introduced into reflectance calculation of two dimensional materials when Interface model is utilized. Thus, it is not correct to use Interface model to solve Fresnel problem of two dimensional materials. Thin film model rather than Interface model can be used to universally solve Fresnel problem of two dimensional materials, and exhibit a better agreement with experimental reflectance results than Interface model. Unexpectedly, on contrary to other remarkable, intriguing properties, two dimensional materials exhibit an ordinary Fresnel optical response, which is same with thin film.

## Introduction

Since the first successful fabrication of graphene in 2004, its electrical, optical and magnetic properties have been widely studied. Reflectance, transmittance, and absorption of materials are also known as materials’ Fresnel problem. Two models are utilized in solving Fresnel problem of graphene. One is that graphene is treated as an infinitesimally thin sheet, which is Interface (IF) model^1–5^; the other is the thin film (TF) model^6–10^, and it is an open question that which one is correct. One of unique features in optical properties of graphene is that its universal absorption in visible band is solely defined by fine structure constant or optical conductivity^1–5^. Derivation for absorption of standing-free graphene (*A* = *πa* = 2.3%, where *A* is absorption, and *a* is fine structure constant) from Stauber was based on IF model. Experimental results agreed well with theoretical simulation in 450–800 nm^1^. However, there was a deviation in other wavelength bands^1^, and the possible reason for this deviation was qualitatively attributed to surface contamination (<450 nm). This discrepancy still cannot be elucidated quantitatively by present available theories. From then on, several theoretical jobs were successfully done to reproduce this special absorption value (*A* = *πa* = 2.3%) from different routes^11–18^. Some jobs were done to study Fresnel optics of two dimensional material (TDM) on the substrate^14,19^, they exhibited different properties as compared to standing-free samples, and they attribute these changes to the substrate effect. It was found that stepwise absorption of two dimensional InAs on the substrate is 1.6% rather than 2.3%, and Fang proposed a formula of (2/(1 + *n*_s_))^2^π*α* (*n*_s_ is refractive index of the substrate) to describe this changes^19^. Although some formulas were proposed based on idea of substrate effect, underlying physical mechanism remains unclear. Until now, formulas of Fresnel Optics for TDM with^14,19^ and without a substrate^11–18^ have been derived, their derivation routes involved IF model^3,17,18^, quantum mechanics^1,11,14–16,19^, and dielectric constant^12,13^. These calculations were complex, scattered, and they need to be summarized into a general theory. In this paper, we treat TDMs as a thin film with a thickness of several Angstrom. In order to solve three questions mentioned above, we attempt to solve Fresnel problem of TDM by TF model based on basic optical coating theory. The origin of IF model is also reexamined. In addition, derived formulas are also utilized to interpret previous experimental results.

## Theory

### Free-standing sample

#### Normal incidence

**Figure 1:**
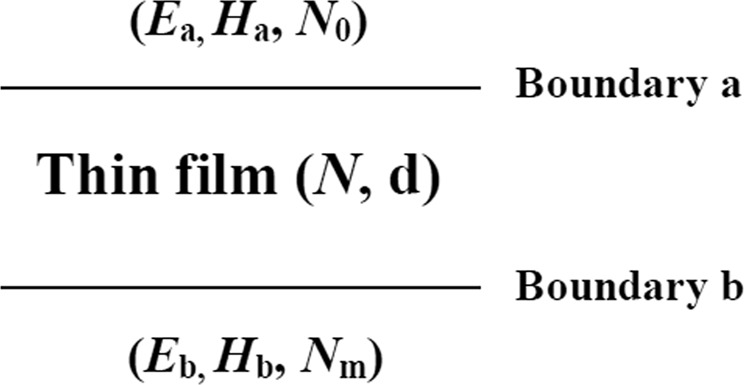
TF model of TDM.

In modern optical coating theory and software, a thin film with a thickness of several Angstrom like graphene also can be treated as a thin film rather than an interface. Here, first we employ basic optical coating theory based on TF model to derive the equation of absorption for TDM at normal incidence. We just give a brief derivation, the details can be found in classical optical books^20–22^. Figure 1 shows TF model of TDM. *E*_a_, *H*_a_, *N*_0,_ are electric field vector, magnetic field vector, and complex refractive index of air, respectively. *N* (*n* − i*k*, *n* is refractive index, and *k* is extinction coefficient), *d*, are the complex refractive index, and thickness of thin film, respectively. *E*_b_, *H*_b_, *N*_m,_ are electric field vector, magnetic field vector, and complex refractive index of the substrate, respectively.

According to boundary condition, electric and magnetic vectors between boundary a and b can be connected in matrix notation, which is shown in Eq. (1), and the first item on the right side of Eq. (1) is characteristic matrix of thin film.

1 $$[\begin{array}{c}{E}_{a}\\ {H}_{a}\end{array}]=[\begin{array}{cc}\cos \,\delta  & (i\,\sin \,\delta )/y\\ iy\,\sin \,\delta  & \cos \,\delta \end{array}]\,[\begin{array}{c}{E}_{b}\\ {H}_{b}\end{array}]$$

where *δ* is phase thickness of thin film, which is defined by Eq. (2), *d* is thickness of thin film, *λ* is wavelength, and *y* is optical admittance of TDM, which is defined by Eq. (3), and *Y* is optical admittance of free space.

2 $$\delta =2\pi Nd/\lambda $$

3 $$y=H/E=NY$$

Equation (1) can be normalized by dividing by *E*_*b*_, Eq. (4) is obtained, where *y*_*m*_ is optical admittance of the substrate, B and C are normalized electric and magnetic vectors.

4 $$[\begin{array}{c}{E}_{a}/{E}_{b}\\ {H}_{a}/{E}_{b}\end{array}]=[\begin{array}{c}B\\ C\end{array}]=[\begin{array}{cc}\cos \,\delta  & (i\,\sin \,\delta )/y\\ iy\,\sin \,\delta  & \cos \,\delta \end{array}]\,[\begin{array}{c}1\\ {y}_{m}\end{array}]$$

*δ* is very small because *d*/*λ* is roughly less than 0.001, so cos*δ* ≈ *1*, *sinδ* ≈ *δ*. Thus, characteristic matrix can be simplified to be Eq. (5).

5 $$[\begin{array}{cc}1 & (i\delta )/y\\ iy\delta  & 1\end{array}]$$

Then, as shown in ref. ^20^, transmittance, reflectance, absorption of TDM can be calculated by Eqs. (6–8), respectively.

6 $$T=\frac{4{y}_{0}\mathrm{Re}({y}_{s})}{({y}_{0}B+C){({y}_{0}B+C)}^{\ast }}$$

7 $$R=(\frac{{y}_{0}B-C}{{y}_{0}B+C}){(\frac{{y}_{0}B-C}{{y}_{0}B+C})}^{\ast }$$

8 $$A=\frac{4{y}_{0}\mathrm{Re}(B{C}^{\ast }-{y}_{s})}{({y}_{0}B+C){({y}_{0}B+C)}^{\ast }}$$

If we set *N*_0_ = *N*_m_ = 1, combine Eqs. (2–5) into (6–8), Eqs. (9–11) are obtained.

9 $${T}_{TF}=\frac{4}{{(2+4\pi nkd/\lambda )}^{2}+4{\pi }^{2}{d}^{2}{({n}^{2}-{k}^{2}+1)}^{2}/{\lambda }^{2}}$$

10 $${R}_{TF}=\frac{{(4\pi nkd/\lambda )}^{2}+4{\pi }^{2}{d}^{2}{(1-{n}^{2}+{k}^{2})}^{2}/{\lambda }^{2}}{{(2+4\pi nkd/\lambda )}^{2}+4{\pi }^{2}{d}^{2}{({n}^{2}-{k}^{2}+1)}^{2}/{\lambda }^{2}}$$

11 $${A}_{TF}=\frac{4\pi nkd/\lambda +4{\pi }^{2}{d}^{2}({n}^{2}-{k}^{2})/{\lambda }^{2}}{{(1+2\pi nkd/\lambda )}^{2}+{\pi }^{2}{d}^{2}({n}^{2}-{k}^{2}+1)/{\lambda }^{2}}$$

For comparison, formulas of transmittance, reflectance, absorption of TDM derived based on IF model are given in Eqs. (12–14)^3^. It should be noted that the term of *4πnkd/λ* is equal to *G/cε*_0_ in equations (50–53) of ref. ^3^, which will be discussed in Section 2.1.3. As shown in Eqs. (9–11) and Eqs. (12–14), the main difference between TF model and IF model is the term including of (n^2^ − k^2^) involved in Eqs. (9–11). There are slight differences in transmittance and absorption between two models, but significant discrepancy in reflectance because the term including of (n^2^ − k^2^) has the same magnitude with (4πnkd/λ)^2^. In other words, eliminating the term including of (n^2^ − k^2^), formulas of TF model reduced to be the ones of IF model. When d/λ is extremely small, this treatment is reasonable for transmittance and absorption, not for reflectance.

12 $${T}_{Interface}=\frac{4}{{(2+4\pi nkd/\lambda )}^{2}}\approx 1-\frac{4\pi nkd}{\lambda }$$

13 $${R}_{Interface}=\frac{{(4\pi nkd/\lambda )}^{2}}{{(2+4\pi nkd/\lambda )}^{2}}$$

14 $${A}_{Interface}=\frac{4\pi nkd/\lambda }{{(1+2\pi nkd/\lambda )}^{2}}\approx \frac{4\pi nkd}{\lambda }$$

**Figure 2:**
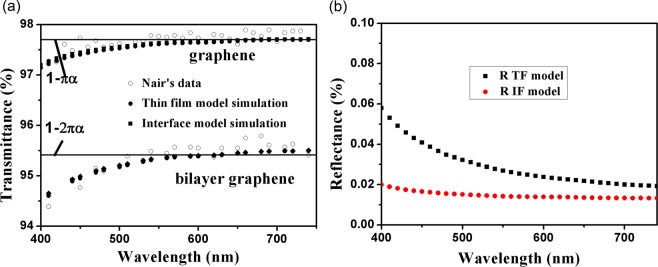
(**a**) Comparison between our calculation and experimental, theoretical results of Nair *(1)*, and (**b**) theoretical reflectance of graphene based on TF model and IF model.

We utilize Eq. (9) of TF model and Eq. (12) of IF model to calculate transmittance of graphene and bilayer graphene, and compare with experimental, theoretical results of Nair^1^. The optical constants of graphene are cited from ref. ^9^ (annealing sample). Figure 2a demonstrates comparison between our calculation and experimental, theoretical results of Nair^1^. Nair’s theoretical calculations were based on IF model, but he ignored wavelength dependence of optical conductance. Here wavelength dependence of optical constant is considered in IF model simulation. TF model and IF model simulation all agree well with experimental results, and even better than Nair’s theoretical prediction. There is no significant difference between TF model and IF model simulation because second term is very small compared with first term in denominator of Eq. (9). Also, there is no significant difference in 600–740 nm among Nair’s theoretical calculation, TF model and IF model simulation because 4πnkd/λ is equivalent to *πα*, which will be discussed in Section 2.1.3. Thus, dispersion of grapheme at <450 nm can answer the first question raised in Introduction section. From Eq. (14), one can see that there is a thickness dependence on the absorption, which can be used to interpret the layer effect of the absorption for the graphene in Fig. 2a. As for deviation from *πα* in 400–450 nm for experiment results in ref. ^1^, the reason is that optical conductance G* is not strictly universal in visible region, and it is wavelength dependence. Several jobs were done to discuss this problem, and optical conductance was described by Eq. (15)^23,24^:

15 $${G}^{\ast }/{G}_{0}=1+C{\alpha }^{\ast }+O({\alpha }^{\ast 2})$$

where α^*^ is a renormalized, dimensionless coupling constant^24^, and *C* is a constant, but there are several values for this debated constant^23,24^. If optical responses of TDM are measured, optical conductance can be accurately determined based on TF model, which will contribute most to precisely study optical conductivity of TDM, and unveil mechanism of unique physical properties of TDM. We also use Eq. (10) of TF model and Eq. (13) of IF model to calculate reflectance of graphene, the results are shown in Fig. 2b. The reflectance calculated is less than 0.1%, which is consistent with experimental results^1^. The calculated reflectance based on TF model is up to three times of the one based on IF model, and this can be explained by the fact that the term including of (*n*^2^ − *k*^2^) has the same magnitude with (4*πnkd/λ*)^2^ in Eq. (10).

#### Oblique incidence

**Figure 3:**
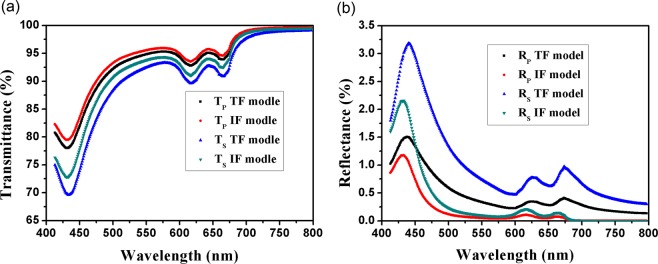
Theoretical p- and s-polarized transmittance (**a**) and reflectance (**b**) of monolayer MoS_2_ based on TF model and IF model.

Fresnel problem of TDM at oblique incidence is discussed based on TF model in ref. ^25^. One can see that refractive angle of TDM is taken into account in our calculation, which significantly differs from theoretical results of Stauber^3,26^. Figure 3 shows theoretical p- and s-polarized transmittance (a) and reflectance (b) of monolayer MoS_2_ based on TF model and IF model at 45° incidence. Compared with normal incidence, besides reflectance, transmittance also shows obvious difference between two models. There are more pronounced differences of s-polarization than p-polarization in transmittance and reflectance. Also, there are significant polarization-independent peak shifts between the two models. For example, in 600–650 nm region, the peaks of both p- and s-polarization curves for IF model are at 616 nm, while the peaks of the curves for TF model are at 625 nm. However, there is no shift in the transmittance curves. The reason can be found by comparison equations (S9, S10, S13, S14) in ref. ^25^ with equations (51, 53, B3) in ref. ^3^. For transmittance, compared with IF model, in TF model, the term, 1/cosθ at p-polarization, or cos^3^θ/cosθ_0_ at s-polarization is added to multiply term of πα/2, and this term just modifies the amplitude, not shifts the peaks. For reflectance, besides the term added multiplying πα/2, another positive term including of (n^2^ − k^2^) is added plus term of π^2^α^2^ in the numerator. Thus, peak reflectance in TF model is red-shifted.

In addition, as shown in equations (S13, S14), s-polarized reflectance and transmittance based on TF model is dependent of incidence angle, which is not same with IF model^3^. However, our formulas for the case of oblique incidence still need further verification of experimental results.

#### 4πnkd/λ

The term of 4πnkd/λ we proposed is readily to be understood and used. Because 4πk/λ is absorption coefficient, we yields another style of approximate absorption of TDM (17, 18):

16 $$A=\alpha dn$$

where *α* is absorption coefficient. Holovský (17, 18) also obtained this result, but he did not give the origin of αdn. It is contradictory that he admitted that the graphene was a thin film with *n*, *k*, and *d*, at the same time he added the value of αdn to formulas derived from IF model. It should be noted that Lee (20) also did initial, qualitatively derivation in discussion of absorption for very thin film, but he did not give final result.

Table 1 summarizes four kinds of approximate formulas for calculation of absorption of TDM^11–18^ in visible and infrared range, and *γ* is layer number. The first formula is equivalent to other three ones only for some special condition (like graphene), the last three formulas are equivalent. Now we derive other three formulas from the term of 4πnkd/λ we proposed.

**Table 1:** Summarization of calculation of absorption for TDM in visible and infrared range.

*Ref*.	^1–5,11,14–18^		^12,13^	This work and^17,18^
*A*	$\gamma \pi a$	$\frac{\sigma d}{{\varepsilon }_{0}c}$	$\frac{\omega d}{c}\text{Im}({\varepsilon }_{r}\text{'})$	$\frac{4\pi nkd}{\lambda }$

Complex refractive index is defined by Eqs. (17) and (18), where *ε*_*r*_ and *μ*_*r*_ are the relative dielectric permittivity and permeability, *σ* is optical conductivity, *ω* is angular frequency, and *ε*’_*r*_ is relative complex dielectric permittivity^18,20,21^.

17 $${N}^{2}={(n-ik)}^{2}={\varepsilon }_{r}{\mu }_{r}-i\frac{{\mu }_{r}\sigma }{\omega {\varepsilon }_{0}}$$

18 $${N}^{2}={(n-ik)}^{2}={\mu }_{r}{\varepsilon }_{r}\text{'}={\mu }_{r}({\varepsilon }_{r}-i\frac{\sigma }{\omega {\varepsilon }_{0}})$$

Derived from Eqs. (17) and (18), optical conductivity and imaginary part of relative complex dielectric permittivity are related to *nk* as shown in Eq. (19), where *ε*_0_ is dielectric permittivity of vacuum, and μ_r_ = 1.

19 $$nk=\frac{{\mu }_{r}\sigma }{2\omega {\varepsilon }_{0}}=\frac{\text{Im}({\varepsilon ^{\prime} }_{r})}{2}$$

Then^17,18^

20 $$A=\frac{4\pi nkd}{\lambda }=\frac{\sigma d}{{\varepsilon }_{0}c}=\frac{G}{{\varepsilon }_{0}c}$$

where *c* is the speed of light in free space, and *G* is optical conductance. For graphene in 600–740 nm:

21 $$G(\omega )\approx {G}_{0}=\frac{\pi {e}^{2}}{2h}=\frac{{e}^{2}}{4\hslash }$$

where *e* is the electron charge, *h* is Plank’s constant, and *ħ* is reduced Plank’s constant. Then

22 $$A=\frac{4\pi nkd}{\lambda }=\frac{\sigma d}{{\varepsilon }_{0}c}=\frac{G}{{\varepsilon }_{0}c}=\frac{{e}^{2}}{4{\varepsilon }_{0}c\hslash }$$

Because the fine structure constant

23 $$\alpha =\frac{{e}^{2}}{4\pi {\varepsilon }_{0}c\hslash }$$

Then

24 $$A=\frac{4\pi nkd}{\lambda }=\frac{\sigma d}{{\varepsilon }_{0}c}=\frac{G}{{\varepsilon }_{0}c}=\frac{{e}^{2}}{4{\varepsilon }_{0}c\hslash }=\pi \alpha $$

Thus, we recover the result of ref. ^1^, this is the reason that absorption in 600–740 nm is same in value between our calculation and Nair’s theoretical prediction.

In addition, combining Eq. (19) into Eq. (14), we yield absorption *A*:

25 $$A=\frac{4\pi nkd}{\lambda }=\frac{\omega d}{c}\text{Im}({\varepsilon ^{\prime} }_{r})$$

We recover the results of refs. ^10,11^.

### On-substrate sample (normal incidence)

Generally, TDM needs to be transferred onto a substrate. Several jobs were done to discuss this issue^14,17–19^, TDM exhibited different properties as compared to standing-free samples, and they attributed these changes to the substrate effect. It was found that stepwise absorption of two dimensional InAs on the substrate is 1.6% rather than 2.3%, and Fang proposed a formula of (2/(1 + *n*_s_))^2^π*α* (*n*_s_ is refractive index of the substrate) to describe this changes^19^. Here we also calculate Fresnel optics of on-substrate TDM. If we set *N*_0_ = 1, combine Eqs. (2–5) into (6–8), respectively, Eqs. (26–28) are obtained, where *n*_*s*_ is refractive index of the substrate.

26 $$A=\frac{4(4\pi nkd/\lambda +4{\pi }^{2}{d}^{2}({n}^{2}-{k}^{2}){n}_{s}/{\lambda }^{2})}{{(1+{n}_{s}+4\pi nkd/\lambda )}^{2}+(4{\pi }^{2}{d}^{2}{({n}^{2}-{k}^{2}+{n}_{s})}^{2}/{\lambda }^{2})}$$

27 $$T=\frac{4{n}_{s}}{{(1+{n}_{s}+4\pi nkd/\lambda )}^{2}+(4{\pi }^{2}{d}^{2}{({n}^{2}-{k}^{2}+{n}_{s})}^{2}/{\lambda }^{2})}$$

28 $$R=\frac{{(1-{n}_{s}-4\pi nkd/\lambda )}^{2}+4{\pi }^{2}{d}^{2}{({n}_{s}-{n}^{2}+{k}^{2})}^{2}/{\lambda }^{2}}{{(1+{n}_{s}+4\pi nkd/\lambda )}^{2}+(4{\pi }^{2}{d}^{2}{({n}^{2}-{k}^{2}+{n}_{s})}^{2}/{\lambda }^{2})}$$

For comparison, formulas of transmittance, reflectance, absorption of TDM derived based on IF model are given in Eqs. (29–31)^3^. Similar to standing-free case, as shown in Eqs. (26–28) and Eqs. (29–31), the main difference between TF model and IF model is the term including of (n^2^ − k^2^) involved in Eqs. (26–28). There are slight differences in transmittance and absorption between two models, but significant discrepancy in reflectance because the term including of (n^2^ − k^2^) has the same magnitude with (4πnkd/λ)^2^. In other words, eliminating the term including of (n^2^ − k^2^), formulas of TF model reduce to be the ones of IF model. When d/λ is extremely small, this treatment is reasonable for transmittance and absorption, not for reflectance.

29 $$A={(\frac{2}{(1+{n}_{s}+4\pi nkd/\lambda )})}^{2}\frac{4\pi nkd}{\lambda }\approx {(\frac{2}{(1+{n}_{s})})}^{2}\frac{4\pi nkd}{\lambda }$$

30 $$T=\frac{4{n}_{s}}{{(1+{n}_{s}+4\pi nkd/\lambda )}^{2}}$$

31 $$R=\frac{{(1-{n}_{s}-4\pi nkd/\lambda )}^{2}}{{(1+{n}_{s}+4\pi nkd/\lambda )}^{2}}$$

**Figure 4:**
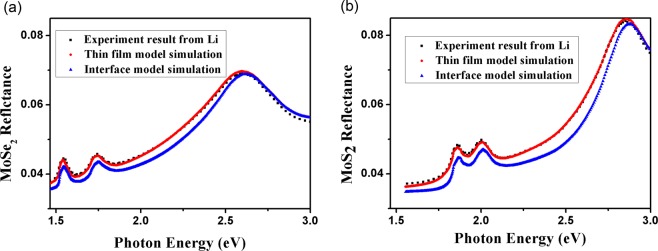
Theoretical simulation and experimental^27^ reflectance results of MoS_2_ (**a**) and MoSe_2_ (**b**) on fused silica substrate.

We utilize TF model and IF model to calculate reflectance of MoS_2_ and MoSe_2_ on fused silica substrate, and optical constants are cited from ref. ^27^. The thicknesses of MoS_2_ and MoSe_2_ are 0.615 nm and 0.646 nm, respectively^27^. Figure 4 shows theoretical and experimental^27^ reflectance results of MoS_2_ (a) and MoSe_2_ (b) on fused silica substrate. TF model simulations exhibit better agreement with experimental results than IF model.

### Origin of IF model

refs. ^3,5,28^ provided derivations for calculation of optical response of TDM based on IF model. In equation (49) of ref. ^3^, *σ* is different from the one in this job. The unit is *Ω*^−1^, and it should be referred to optical conductance, not optical conductivity (the unit of *Ω*^−1^.*m*^−1^) in this job. While in ref. ^5^ and Eq. (8) of ref. ^28^, *σ* is same with the one in this job. The relationship between *σ*_*IF*_ in IF model and *σ*_*TF*_ is described in Eq. (32)^26^. We can see that in refs. ^3,5,28^, they all took thickness of TDM into account of their calculation of electric current density *j*, which is contradicted with the assumption that TDM is an interface. Thus, in essence, IF model is also an approximate style of TF model. A larger error is introduced into reflectance calculation when IF model is utilized. Thus, TF model rather than IF model is an accurate way to solve Fresnel problem of TDM.

32 $${\sigma }_{{\rm{IF}}}={\sigma }_{{\rm{TF}}}d$$

## Conclusion

It should be noted that when *d/λ* is not extremely small, in other word, the wavelength extend to ultraviolet and X ray range, Eq. (5) is not correct, an accurate form of first term on the right side of Eq. (1) must be used. Our job does not deny the fact that there are many fantastic optical properties existing in multilayer TDM, such as interaction between bilayer graphene. These new properties will give some small corrections to optical conductivity, which can be macroscopically characterized by optical constants (n and k). There are no contradiction between our job and new optical properties.

Alternatively, it is found that the product of refractive index *n*, absorption coefficient *a*, thickness *d*, plays a key role in the approximate calculation of absorption of TDM at normal incidence in visible and infrared range. Previous calculation of Fresnel optics for TDM (standing-free and on-substrate samples) all can be recovered from our derived formulas by eliminating the term of (*n*^2^ − *k*^2^) at normal incidence, and they are just approximate results of our calculation based on TF model. IF model, in essence, is a kind of approximate TF model, and the main difference between two models is the term of (*n*^2^ − *k*^2^) involved in TF model at normal incidence. A significant error is introduced into reflectance calculation of two dimensional materials when IF model is utilized. The differences between two models are complicated at oblique incidence, which needs further experimental verification. IF model is not suitable for solve Fresnel problem of two dimensional materials. Our job reveals that TDM is a thin film rather than an interface or a boundary. It indicates that basic optical coating theory and traditional optical software apply to TDM, and our job will pave the way for widely application of TDM in optical coating field. Especially, our job will contribute most to design novel filters based on TDM multilayer that was referred to as van der Waals heterostructures in refs. ^29,30^.

## Supplementary information

Supplementary information
