# Supplementary material for: Origin of Fresnel problem of two dimensional materials
Source: Sci Rep. 2019 Nov 28;9:17825. doi: 10.1038/s41598-019-54338-0 (PMC6882841; doi:10.1038/s41598-019-54338-0)
Supplement: Supplementary file 1 — Supplementary information [file 41598_2019_54338_MOESM1_ESM.doc]

Supplementary Materials for

Origin of Fresnel problem of two dimensional materials

Xiaodong Wang*, Bo Chen

correspondence to: [wangxiaodong@ciomp.ac.cn](mailto:wangxiaodong@ciomp.ac.cn)

**This PDF file includes:**

Supplementary Text

Figs.S1

Supplementary Text

Calculation at oblique incidence

Figure S1 shows thin film model of TDM at oblique incidence.

Oblique admittances for p- and s-polarized lights are introduced (1):

$\eta_{p}=\frac{y}{\cos\theta}=\frac{NY}{\cos\theta}$ (for p-polarized light ) (S1)

$\eta_{s}=y\cos\theta=NY\cos\theta$ (for s-polarized light) (S2)

Characteristic Matrix can be normalized:

$$\left[\begin{matrix} E_{a}/E_{b} \\ H_{a}/E_{b} \end{matrix}\right]=\left[\begin{matrix} B \\ C \end{matrix}\right]=\left[\begin{matrix} \cos\delta& (i\sin\delta)/\eta\\ i\eta\sin\delta& \cos\delta\end{matrix}\right]\left[\begin{matrix} 1 \\ \eta_{m} \end{matrix}\right]$$

(S3)

Where *δ* is defined:

$\delta=2\pi Nd\cos\theta/\lambda$(S4)

Because *d/λ* is extremely small in visible region, so cos*δ≈1, sinδ≈δ.* Thus, Characteristic Matrix is reduced to be:

$$\left[\begin{matrix} 1 & (i\delta)/\eta\\ i\eta\delta& 1 \end{matrix}\right]$$

(S5)

Then, as shown in Ref. S1, transmittance, reflectance, absorption of TDM can be calculated by Equation (S6), (S7), and (S8), respectively.

$$A=\frac{4\eta_{0}\mathrm{Re}(BC^{*}-\eta_{m})}{(\eta_{0}B+C)(\eta_{0}B+C)^{*}}$$

(S6)

$$T=\frac{4y_{0}\mathrm{Re}(\eta_{m})}{(\eta_{0}B+C)(\eta_{0}B+C)^{*}}$$

(S7)

$$R=\left(\frac{\eta_{0}B-C}{\eta_{0}B+C}\right)\left(\frac{\eta_{0}B-C}{\eta_{0}B+C}\right)^{*}$$

(S8)

$T=\frac{\frac{4}{\cos\theta_{0}^{2}}}{\left(\frac{4\pi nkd}{\lambda\cos\theta}+\frac{2}{\cos\theta_{0}}\right)^{2}+\frac{4\pi^{2}d^{2}}{\lambda^{2}}\left(\frac{\cos\theta}{\cos\theta_{0}^{2}}+\frac{n^{2}-k^{2}}{\cos\theta}\right)^{2}}\approx\frac{\frac{4}{\cos\theta_{0}^{2}}}{\left(\frac{4\pi nkd}{\lambda\cos\theta}+\frac{2}{\cos\theta_{0}}\right)^{2}}$For simplicity, we only perform calculation for free-standing case. We set *N*_0_=*N*_m_, cos*θ*_0_=cos*θ*_m_, and insert Equations (S1, S3-S5) into Equation (S6-S8), Equations (S9-S11) are obtained for p-polarized light:

(S9)

$$R=\frac{\left(\frac{4\pi nkd}{\lambda\cos\theta}\right)^{2}+\frac{4\pi^{2}d^{2}}{\lambda^{2}}\left(\frac{\cos\theta}{\cos\theta_{0}^{2}}-\frac{n^{2}-k^{2}}{\cos\theta}\right)^{2}}{\left(\frac{4\pi nkd}{\lambda\cos\theta}+\frac{2}{\cos\theta_{0}}\right)^{2}+\frac{4\pi^{2}d^{2}}{\lambda^{2}}\left(\frac{\cos\theta}{\cos\theta_{0}^{2}}+\frac{n^{2}-k^{2}}{\cos\theta}\right)^{2}}=\frac{\left(\frac{4\pi nkd}{\lambda\cos\theta}\right)^{2}+\frac{4\pi^{2}d^{2}}{\lambda^{2}}\left(\frac{\cos\theta}{\cos\theta_{0}^{2}}-\frac{n^{2}-k^{2}}{\cos\theta}\right)^{2}}{\left(\frac{4\pi nkd}{\lambda\cos\theta}+\frac{2}{\cos\theta_{0}}\right)^{2}}$$

(S10)

$$A=\frac{\frac{4}{\cos\theta_{0}}\left(\frac{4\pi nkd}{\lambda\cos\theta}+\frac{4\pi^{2}d^{2}}{\lambda^{2}\cos\theta_{0}}\left(n^{2}-k^{2}\right)\right)}{\left(\frac{4\pi nkd}{\lambda\cos\theta}+\frac{2}{\cos\theta_{0}}\right)^{2}+\frac{4\pi^{2}d^{2}}{\lambda^{2}}\left(\frac{\cos\theta}{\cos\theta_{0}^{2}}+\frac{n^{2}-k^{2}}{\cos\theta}\right)^{2}}\approx\frac{\frac{4}{\cos\theta_{0}}\times\frac{4\pi nkd}{\lambda\cos\theta}}{\left(\frac{4\pi nkd}{\lambda\cos\theta}+\frac{2}{\cos\theta_{0}}\right)^{2}}$$

(S11)

We set *N*_0_=*N*_m_, cos*θ*_0_=cos*θ*_m_, and insert Equations (S2-S5) into Equation (S6-S8), Equations (S12-S14) are obtained for s-polarized light:

$$A=\frac{4\cos\theta_{0}\left(\frac{4\pi nkd\cos^{3}\theta}{\lambda}+\frac{4\pi^{2}d^{2}\left(n^{2}-k^{2}\right)\cos^{4}\theta\cos\theta_{0}}{\lambda^{2}}\right)}{\left(2\cos\theta_{0}+\frac{4\pi nkd\cos^{3}\theta}{\lambda}\right)^{2}+\frac{4\pi^{2}d^{2}\cos^{2}\theta}{\lambda^{2}}\left(\cos\theta_{0}+\left(n^{2}-k^{2}\right)\cos^{2}\theta\right)^{2}}\approx\frac{4\cos\theta_{0}\left(\frac{4\pi nkd\cos^{3}\theta}{\lambda}\right)}{\left(2\cos\theta_{0}+\frac{4\pi nkd\cos^{3}\theta}{\lambda}\right)^{2}}$$

(S12)

$$T=\frac{4\cos\theta_{0}^{2}}{\left(2\cos\theta_{0}+\frac{4\pi nkd\cos^{3}\theta}{\lambda}\right)^{2}+\frac{4\pi^{2}d^{2}\cos^{2}\theta}{\lambda^{2}}\left(\cos\theta_{0}+\left(n^{2}-k^{2}\right)\cos^{2}\theta\right)^{2}}\approx\frac{4\cos\theta_{0}^{2}}{\left(2\cos\theta_{0}+\frac{4\pi nkd\cos^{3}\theta}{\lambda}\right)^{2}}$$

(S13)

$$R=\frac{\left(\frac{4\pi nkd\cos^{3}\theta}{\lambda}\right)^{2}+\frac{4\pi^{2}d^{2}\cos^{2}\theta}{\lambda^{2}}\left(\cos\theta_{0}-\left(n^{2}-k^{2}\right)\cos^{2}\theta\right)^{2}}{\left(2\cos\theta_{0}+\frac{4\pi nkd\cos^{3}\theta}{\lambda}\right)^{2}+\frac{4\pi^{2}d^{2}\cos^{2}\theta}{\lambda^{2}}\left(\cos\theta_{0}+\left(n^{2}-k^{2}\right)\cos^{2}\theta\right)^{2}}=\frac{\left(\frac{4\pi nkd\cos^{3}\theta}{\lambda}\right)^{2}+\frac{4\pi^{2}d^{2}\cos^{2}\theta}{\lambda^{2}}\left(\cos\theta_{0}-\left(n^{2}-k^{2}\right)\cos^{2}\theta\right)^{2}}{\left(2\cos\theta_{0}+\frac{4\pi nkd\cos^{3}\theta}{\lambda}\right)^{2}}$$

(S14)

Reference

S1. H. A. Macleod, *Thin-Film Optical Filters* (CRC Press, ed.4, 2010).


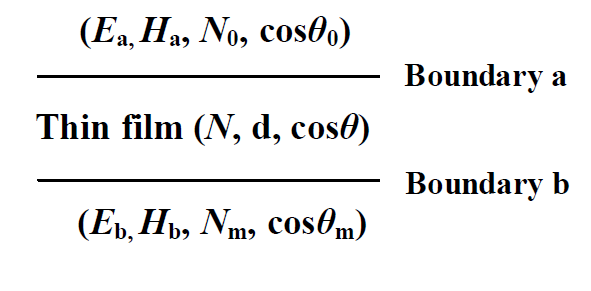


Fig. S1. TF model of TDM at oblique incidence.
